# Late gadolinium enhancement on cardiac magnetic resonance combined with ^123^I- metaiodobenzylguanidine scintigraphy strongly predicts long-term clinical outcome in patients with dilated cardiomyopathy

**DOI:** 10.1371/journal.pone.0217865

**Published:** 2019-06-20

**Authors:** Misato Chimura, Shinichiro Yamada, Yasuyo Taniguchi, Yoshinori Yasaka, Hiroya Kawai

**Affiliations:** Himeji Cardiovascular Center, Himeji, Japan; Scuola Superiore Sant'Anna, ITALY

## Abstract

Late gadolinium enhancement (LGE) on cardiac magnetic resonance (CMR) is limited in its ability to detect diffuse interstitial fibrosis, which is commonly found in idiopathic dilated cardiomyopathy (DCM). On the other hand, Washout rate (WR) by cardiac ^123^I- metaiodobenzylguanidine (^123^I-MIBG) scintigraphy which evaluates cardiac sympathetic nervous function, is a useful tool for predicting the prognosis in DCM. We investigated the predictive value of the combination of two different types of examinations, LGE on CMR and WR by ^123^I-MIBG scintigraphy for outcomes in DCM compared with LGE alone. One-hundred forty-eight DCM patients underwent CMR and ^123^I-MIBG scintigraphy. Patients were divided into 4 groups according to the presence or absence of LGE and WR cut-off value of 45% for predicting prognosis based on receiver operating characteristic curve analysis. Cardiac deaths, re-hospitalization for heart failure, implantation of a left ventricular assist device, and life-threatening ventricular arrhythmias were defined as clinical events. Forty-two DCM patients reached the clinical events during the median follow-up for 9.1 years (interquartile range, 8.0–9.2 years).Multivariable Cox regression analysis identified WR≥45%+LGE positive group as an independent predictor of cardiac events (HR 3.18, 95%CI 1.36–7.45, p = 0.008). Notably, there was no significance in the cardiac event-free survival rate between the WR<45%+LGE positive and WR≥45%+LGE negative groups (p = 0.89). The combination of WR by ^123^I-MIBG scintigraphy and LGE on CMR, which evaluate different type of cardiac deterioration, serves as a stronger predictor of long-term outcomes in DCM patients than LGE alone.

## Introduction

Cardiac magnetic resonance (CMR) is well established as the reference imaging method for the assessment of cardiac anatomy and function [1]. The late gadolinium enhancement (LGE) on CMR by using gadolinium contrast agents, evaluates the myocardial properties and provides the prognostic information about nonischemic cardiomyopathy. However, in LGE on CMR image contrast relies on the difference in signal intensity between normal and fibrotic myocardium, so it is difficult to assess the diffuse interstitial fibrosis which is a characteristic fibrotic pattern of dilated cardiomyopathy (DCM) [2]. Therefore the high risk DCM patients for cardiac events may have been missed in evaluating the presence of LGE alone.

Cardiac imaging with ^123^I-metaiodobenzylguanidine (^123^I-MIBG), an analogue of norepinephrine, is a useful tool for detecting abnormal cardiac sympathetic nervous activity in heart failure (HF) patients [3–6]. Increased sympathetic nervous activity in DCM patients is shown to be associated with a poor prognosis [7–9]

We hypothesized that the combination of two different types of examinations, LGE on CMR and ^123^I-MIBG scintigraphy, may provide more prognostic information in DCM patients than LGE on CMR alone. The aim of this study was to classify the relationship between LGE on CMR and the findings of ^123^I-MIBG scintigraphy and to evaluate the predictive value of the combination of two modalities for cardiac events in DCM patients.

## Methods

### Patients

**Fig 1:**
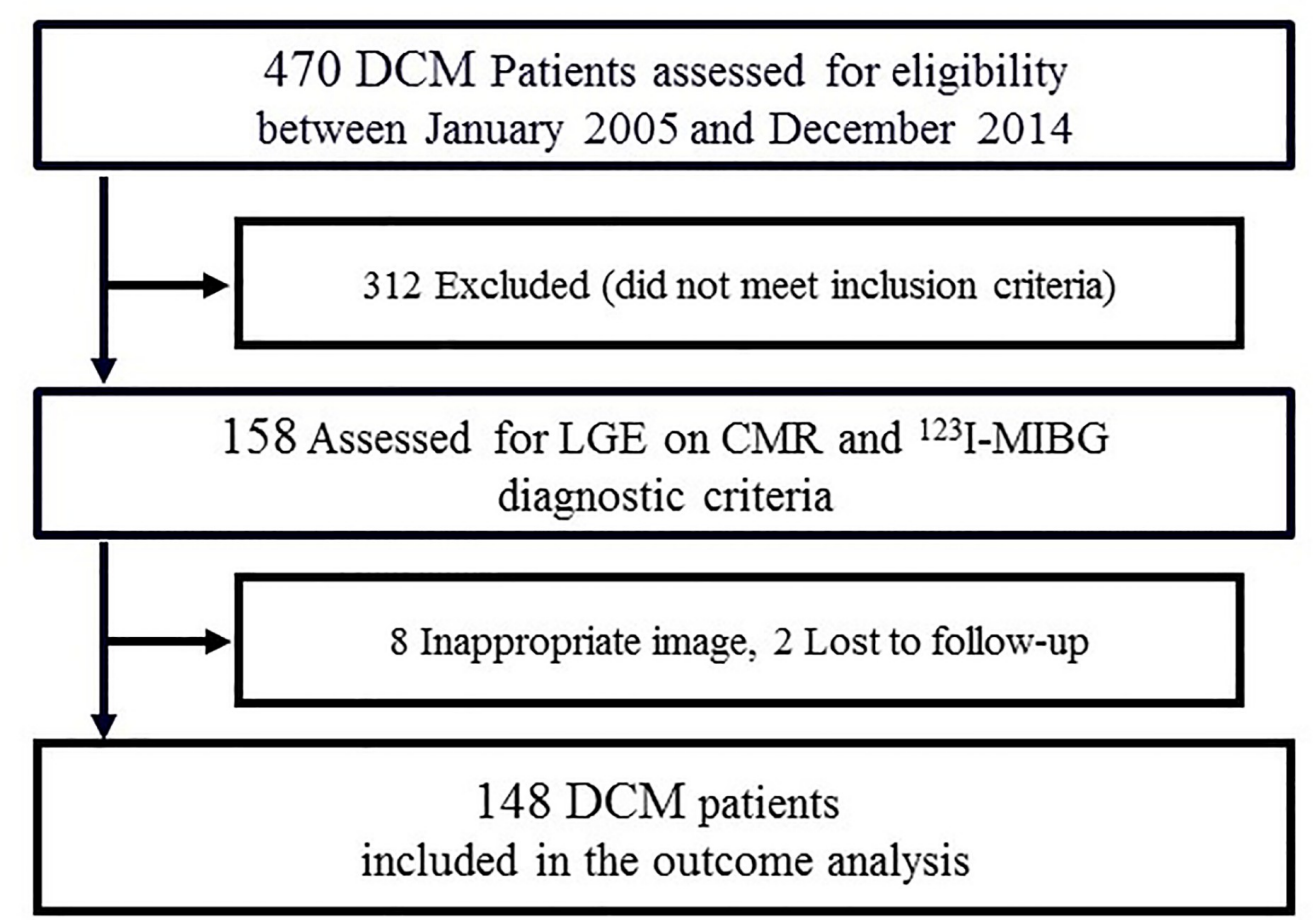
Patient flow chart. Out of 470 patients, 312 patients who did not meet inclusion criteria, 8 patients who had inappropriate images and 2 patients who were lost to follow-up were excluded, resulting in a final cohort of 148 patients.

We conducted a longitudinal study in a cohort of consecutive 470 DCM patients who were referred to Himeji Cardiovascular Center with HF at their initial visit between January 2005 and December 2014. Patients were excluded before entry if they had had any of the following: Contraindications to CMR (significant renal dysfunction [glomerular filtration rate of ≤30 mL·min^-1^·1.73 m^-2^], or implanted devices such as pacemakers and/or defibrillators), neuro-degenerative diseases such as Parkinson disease and dementia with Lewy bodies, and receiving reserpine or tricyclic antidepressants. The remaining 158 DCM patients underwent CMR and ^123^I-MIBG scintigraphy (Fig 1). The diagnosis of DCM was made according to the criteria of the World Health Organization/International Society and Federation of Cardiology [10]. Coronary angiography was performed in all DCM patients to exclude significant stenoses of the coronary arteries. Both CMR and ^123^I-MIBG scintigraphy were performed within 1 month when they became clinically stable with medications. Baseline clinical variables included New York Heart Association (NYHA) functional class, serum creatinine level, plasma B-type natriuretic peptide (BNP) level, and prescribed medication for HF. The study conformed with the principles outlined in the Declaration of Helsinki. This study was approved by the research ethics committee of Himeji Cardiovascular Center and carried out in accordance with approved guidelines. For this analysis of clinically acquired data, the institutional review board waived the need for patients’ written informed consent.

### CMR image acquisition

**Fig 2:**
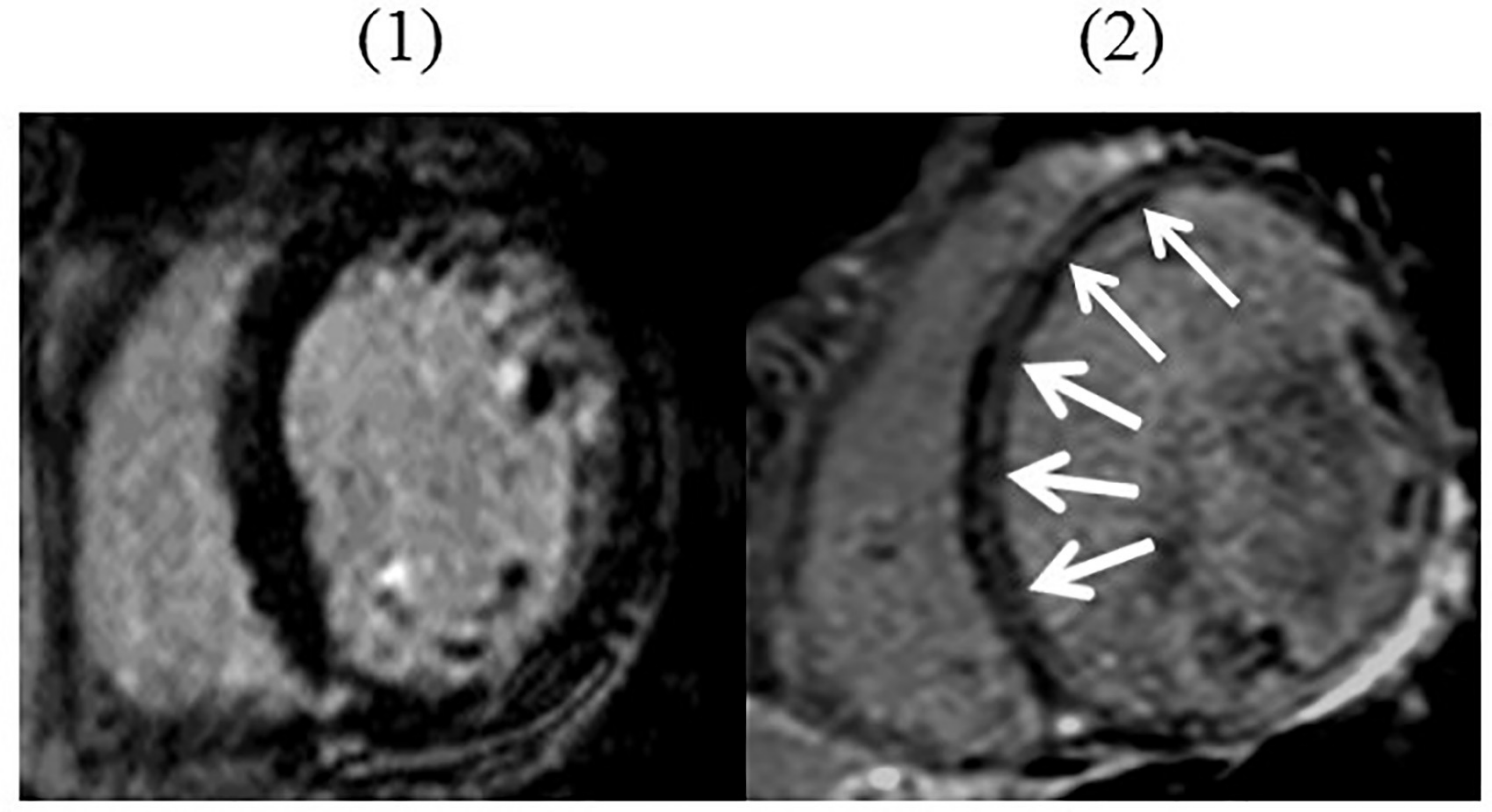
Representative cases of CMR. (1) Cardiac magnetic resonance (CMR) images in midventricular short axis views did not show late gadolinium enhancement (LGE). (2) LGE demonstrated a near-circumferential pattern of midwall LGE (white arrows) in the anterior, septal, inferior, and inferolateral segments at midventricular short axis.

CMR imaging was performed using a 1.5-T Intera Achieva scanner (Philips Medical Systems, Best, the Netherlands) and a standardized protocol. Cine images were acquired with a steady-state, free-precession sequence in long-axis planes and contiguous short-axis slices from the atrioventricular ring to the apex, as previously described [11]. Ten minutes after intravenous injection of 0.1 mmol/kg gadolinium-DTPA (Schering AG, Berlin, Germany), LGE images were obtained by using an inversion-recovery gradient echo sequence in identical long-axis and short-axis planes. Inversion times were optimized to null normal myocardium, and images were repeated in 2 separate phase-encoding directions to exclude artefacts [12]. LGE was only deemed to be present when the area of signal enhancement could be seen in both phase-swapped images and in a cross-cut long-axis image obtained by the independent observers (Fig 2). The LGE was assessed visually by 2 independent readers blinded to all patient details.

### Imaging and analysis of myocardial ^123^I-MIBG scintigraphy

A complete imaging protocol included planar and SPECT images obtained at 15 min (early) and 3 hours (delayed) after intravenous injection of 111 MBq (3 mCi) at rest. For the gamma camera, PRISM-IRIX (Shimadzu Corp, Kyoto, Japan), with 3 heads, attached to a low-energy, high-resolution collimator, and then stored in 256×256 matrix. The data were analyzed by two independent observers blinded to patients’ clinical conditions. On anterior planar images, regions of interest (ROIs) were drawn manually over the whole heart (H) and upper mediastinum (M) was used as the reference background. For the quantitative analysis of ^123^I-MIBG, the delayed heart to mediastinum ratio (H/M) and washout rate (WR) were calculated as described in a previous study [13].

### Transthoracic echocardiography

Comprehensive transthoracic echocardiography was performed by highly experienced research sonographers by using commercially available Aplio (Toshiba Medical Systems, Tokyo, Japan).

Two-dimensional and color Doppler echocardiography were performed in standard parasternal and apical views. LV end-diastolic volume (EDV), end-systolic volume (ESV), and LV ejection fraction (EF) were measured using a modified Simpson method.

### Follow-up and end points

All patients were followed for nonfatal events by outpatient clinic or telephone and by medical record review. Cardiac deaths, re-hospitalization for HF, implantation of an LV assist device, and life-threatening ventricular arrhythmia events were defined as cardiac events building the composite end points. Life threatening arrhythmias were defined as ventricular fibrillation (VF) and sustained ventricular tachycardia (VT), which lasted more than 30 seconds, at R–R intervals of ≤ 400 milliseconds.

### Statistical analysis

Data are expressed as mean ± standard deviation for continuous variables, and median and interquartile range for non-normally distributed continuous variables. Categorical variables were compared using the x^2^ test. Continuous variables were compared using a one-way analysis of variance with Bonferroni correction or the Steel–Dwass test for multiple unadjusted comparisons when appropriate after the assessment of normal distribution. Data from the findings of ^123^I-MIBG scintigraphy between the presence and absence of LGE were compared by using the Wilcoxon–Mann–Whitney test. WR by ^123^I-MIBG scintigraphy cut-off values were determined based on receiver operating characteristics (ROC) analysis. Univariate and multivariate Cox proportional hazards models were performed to adjust for differences in baseline characteristics or pertinent covariates on outcomes. Multivariable Cox regression analysis was performed using covariates that significantly predicted adverse cardiac events in univariate analysis. Event-free survival curves were drawn according to the Kaplan-Meier method, and the comparison among curves was carried out by the log-rank test. Two-tailed p values of <0.05 were considered statistically significant. MedCalc Version 12.7.8 (Acacialaan 22, B-8400 Ostend, Belgium) was used for all analyses.

## Results

Of 158 DCM patients, 8 with inappropriate CMR images and 2 who were lost to follow-up were excluded, resulting in a final cohort of 148 patients (Fig 1). Cardiac events occurred in 42(28%) DCM patients including 12 cardiac deaths, 2 implantations of LV assist device and 28 re-hospitalizations including 10 life-threatening arrhythmic events during a median follow-up of 9.1 years (interquartile range, 8.0–9.2 years). According to the ROC curve analysis, the WR cut-off value for developing cardiac events was 45%. In response to this result, we divided all 148 DCM patients into 4 groups with the WR cut-off value and the presence or absence of LGE as follows: WR<45%+LGE negative (n = 18), WR≥45%+LGE negative (n = 30), WR<45%+LGE positive (n = 37) and WR ≥45%+LGE positive (n = 63) (Table 1).

**Table 1:** Patients characteristics.

	All patients	WR<45%+ LGE negative	WR≥45%+ LGE negative	WR<45%+ LGE positive	WR≥45%+ LGE positive	p Value
	(n = 148)	(n = 18)	(n = 30)	(n = 37)	(n = 63)	
***General information***						
Age, years	58.7 ± 14.9	53.1 ± 14.8	58.9 ± 12.9	58.4 ± 15.5	60.4 ± 15.4	0.35
Male, n (%)	93 (63)	13 (72)	18 (60)	24 (65)	38 (60)	0.8
NYHA functional class Ⅰ/Ⅱ/Ⅲ/Ⅳ	8 / 46 / 91 / 3	3 / 9 / 6 / 0	2 / 8 / 20 / 0	2 / 12 / 22/ 1	1 / 17 / 43 / 2	<0.01
Family history of DCM, n (%)	19 (13)	1 (6)	3 (10)	8 (22)	7 (11)	0.3
Atrial fibrillation, n (%)	49 (33)	3 (16)	9 (30)	13 (35)	24 (38)	0.38
Systolic blood pressure, mmHg	118 ± 19	120 ± 19	125 ± 18	114 ± 17	117 ± 22	0.25
***ECG data***						
Heart rate, beats/min	77 ± 17	76 ± 13	73 ± 14	78 ± 18	78 ± 19	0.56
QRS duration, msec	121 ± 32	118 ± 29	117 ± 33	120 ± 36	125± 31	0.66
***Laboratory data***						
Creatinine, mg/dL	0.84 ± 0.25	0.81 ± 0.18	0.83 ± 0.21	0.85 ± 0.24	0.89 ± 0.40	0.88
BNP, pg/mL	309 (237–430)	98 (10–322)	296 (104–570)	339 (128–954)	381 (154–1068)	<0.01
***Medications***						
ACEI and/ or ARB, n (%)	141 (95)	18 (100)	28 (93)	34 (92)	61 (97)	0.5
Beta-blockers, n (%)	146 (99)	18 (100)	30 (100)	37 (100)	61 (97)	0.44
Diuretics, n (%)	62 (42)	7 (39)	9 (31)	22 (59)	24 (38)	0.09
MRA, n (%)	41 (28)	7 (39)	6 (20)	9 (27)	19 (30)	0.55
Digoxin, n (%)	15 (10)	1 (6)	1 (3)	5 (14)	8 (13)	0.42
Amiodarone, n (%)	1 (2)	0 (0)	0 (0)	0 (0)	1 (2)	0.72
***Echocardiography***						
LVEDV, ml	210 ± 61	198 ± 52	194 ± 50	215 ± 63	217 ± 66	0.27
LVESV, ml	146 ± 56	134 ± 46	132 ± 50	151 ± 61	154 ± 59	0.26
LVEF, %	30.8 ± 7.9	33.5 ± 7.1	30.6 ± 8.5	30.5 ± 8.0	30.3 ± 7.9	0.5
^***123***^***I-MIBG scintigraphy data***						
delayed H/M	1.85 ± 0.40	1.95 ± 0.34	1.92 ± 0.44	1.82 ± 0.45	1.80 ± 0.38	0.36
washout rate, %	48 ± 13	34 ± 7	56 ± 7	34 ± 9	55 ± 7	<0.01

ARB, angiotensin II receptor blocker; ACEI, angiotensin converting enzyme inhibitor; BNP, B-type natriuretic peptide; BP, blood pressure; DCM, dilated cardiomyopathy; ECG, Electrocardiogram; H/M ratio, heart to mediastinum ratio; LGE, late gadolinium enhancement; LVEF, left ventricular ejection fraction; LVEDV, left ventricular end-diastolic volume; LVESV, left ventricular end-systolic volume; MRA, mineralocorticoid receptor antagonists; NYHA, New York Heart Association; and　^123^I-MIBG, ^123^-I metaiodobenzylguanidine scintigraphy;

### Clinical characteristics and LGE and WR

Table 1 showed the characteristics of DCM patients among 4 groups. The mean age was 59 ± 15 years. Almost all the patients belonged to NYHA classes II or III. LGE was observed in 100 (68%) of 148 DCM patients. Of them, 74 patients (74%) had midwall LGE, 21 (21%) diffuse LGE and 5(5%) focal LGE. There were no significant difference in clinical characteristics and prescribed medication for HF among 4 groups, except for NYHA functional class and BNP level. WR<45% +LGE negative patients had lowest BNP level among 4 groups, but delayed H/M was similar among them.

### The relationship between cardiac ^123^I-MIBG scintigraphic findings and LGE on CMR

**Fig 3:**
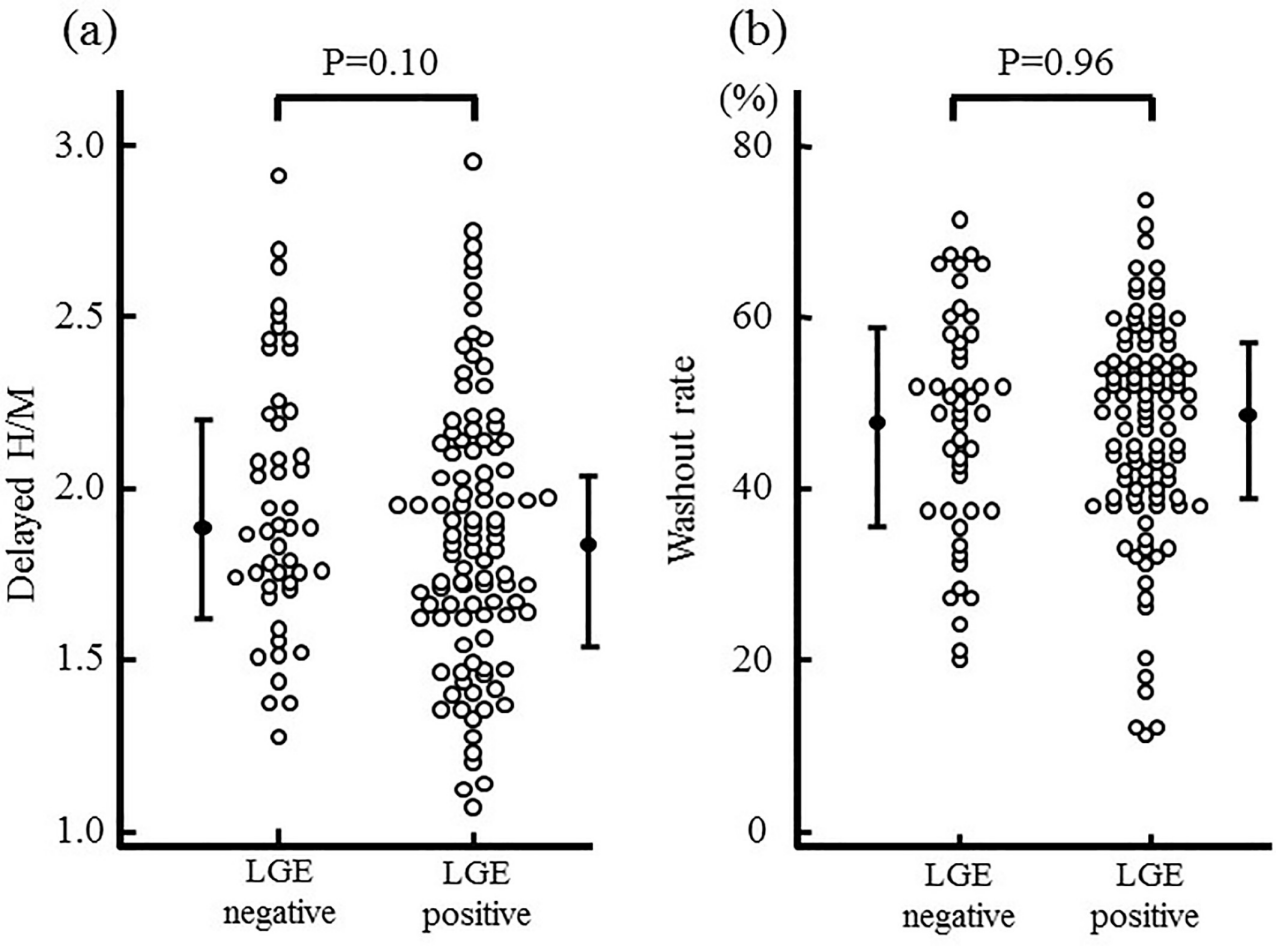
Comparison of cardiac ^123^I-MIBG scintigraphic findings between the presence or absence of LGE. The delayed H/M (a) and WR (b) were not significantly different between the presence and absence of LGE.

The relationship between cardiac ^123^I-MIBG scintigraphic findings, such as delayed H/M and WR, and the presence or absence of LGE is shown in Fig 3.The delayed H/M did not differ significantly between the presence and absence of LGE (1.93±0.40 vs.1.81±0.40, p = 0.10). WR was higher than the control [14] both groups and the difference was not statistically significant between two groups. (48 ± 13% vs.48 ± 13%,p = 0.90).

### Prognostic value of the combination of LGE and WR

**Fig 4:**
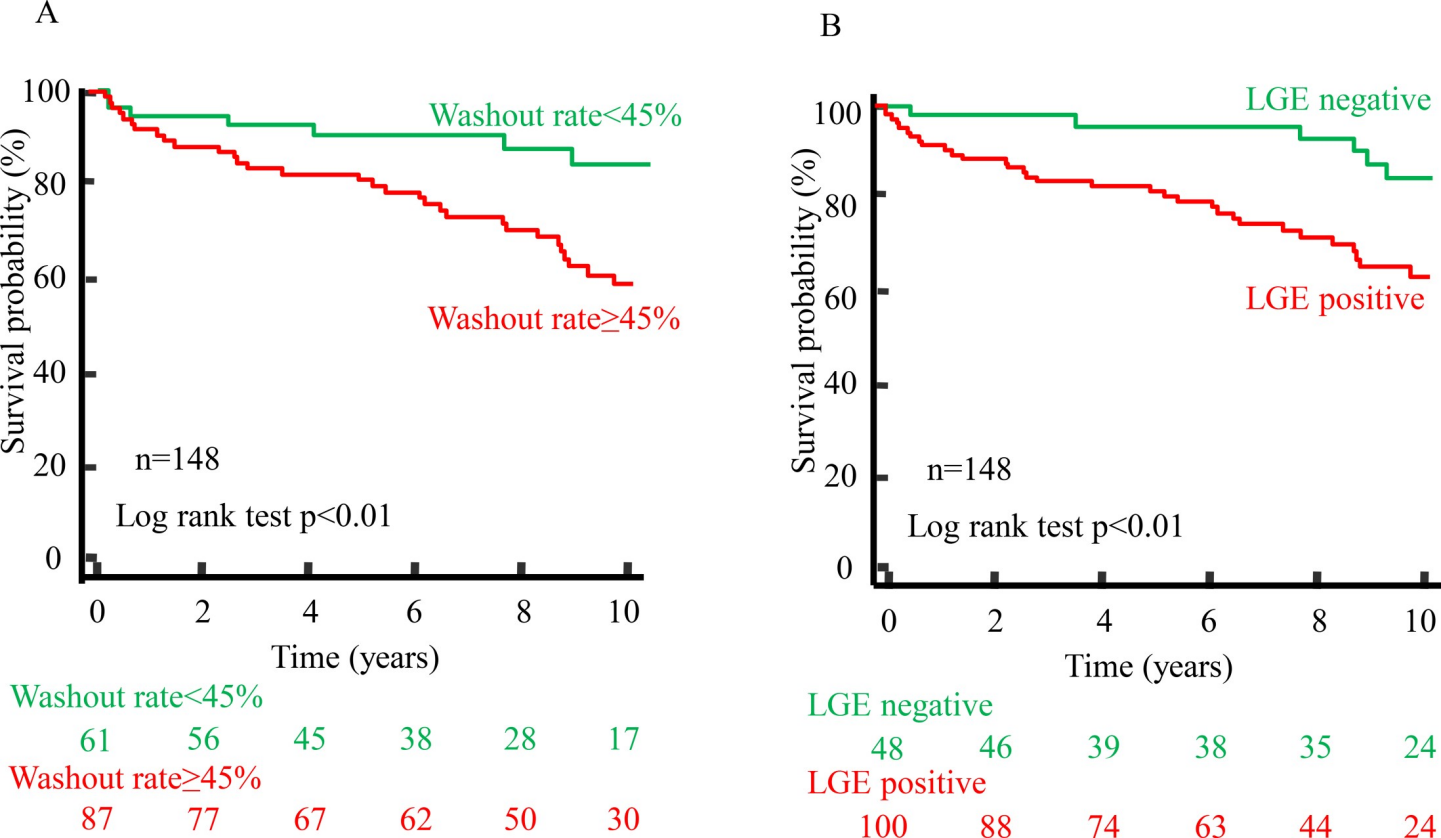
Kaplan-Meier analysis according to LGE on CMR and WR by ^123^I-MIBG scintigraphy. (A) DCM patients with WR ≥ 45% (red line) had a worse outcome than those with WR<45% (green line). (B) DCM patients with LGE positive (red line) had a worse outcome than those with LGE negative (green line).

**Fig 5:**
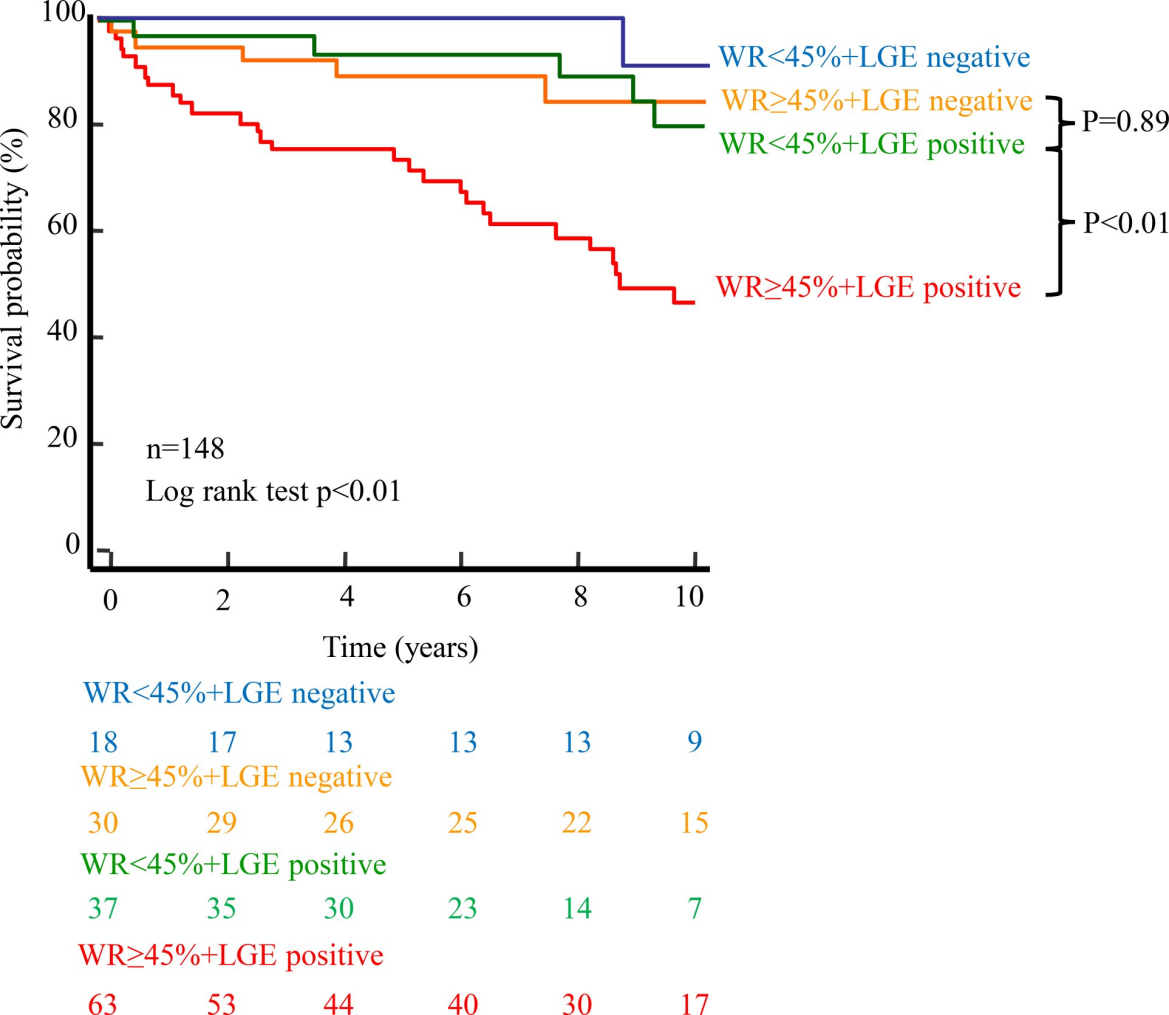
Kaplan-Meier analysis of the combination with LGE on CMR and WR by ^123^I-MIBG scintigraphy. The WR ≥45%+LGE positive group had the worst prognosis. Of note, the event rate in the WR<45% + LGE positive and WR ≥45%+LGE negative groups were intermediate (p = 0.89).

Of 148 DCM patients, 42 patients experienced cardiac events. Among the 4 groups, 30 (48%) of the 63 WR ≥45%+LGE positive patients experienced cardiac events. On the other hand, 5 (14%) of the 37 WR<45%+LGE positive patients and 6 (20%) of the 31 WR≥45%+LGE negative patients experienced them. Only 1 (5%) of the 18 WR<45% +LGE negative patients experienced a cardiac event. Kaplan-Meier analysis demonstrated that WR≥45% and the LGE positive patients were significantly associated with cardiac events, respectively (p < 0.01, Fig 4A and 4B). Among the 4 groups, Kaplan-Meier analysis showed that the WR≥45% +LGE positive group had the worst outcome (p < 0.05). Notably, there was no significant difference in the cardiac event-free survival rate between the WR<45%+LGE positive and WR≥45%+LGE negative groups (p = 0.89). (Fig 5)

We evaluated the following parameters to determine the association of their baseline value with cardiac events using Cox proportional hazard regression analyses: age, gender, NYHA functional class, Atrial fibrillation, systolic blood pressure, BNP level, creatine level, heart rate, QRS duration, LVEDV, LVESV, LVEF, delayed H/M, WR≥45% and the presence of LGE. Univariate Cox proportional hazard regression analysis revealed that BNP level, creatine level, delayed H/M, WR≥45% and the presence of LGE were significantly associated with cardiac events (p<0.05)(Table 2). On a stepwise multivariable Cox model adjusted for factors that were significant in the univariable analysis, the BNP level, the presence of LGE and WR≥45% were significant independent predictors for all cardiac events. On an alternative multivariable Cox regression analysis, the significant association of WR≥45%+ LGE-positive group with further cardiac events remained even after adjusting for the BNP level. (Table 3, Model 2, Model 3 and Model 4).

**Table 2:** Univariate Cox regression analyses for predicting outcome.

	Univariate	
	HR (95% CI)	p Value
Age, per 1 year increase	1.02 (0.99–1.04)	0.11
Male	0.68 (0.37–1.27)	0.23
NYHA functional class	1.81 (0.97–3.39)	0.06
Atrial fibrillation	1.69 (0.91–3.15)	0.1
Systolic BP, per 10 mmHg decrease	1.02 (0.99–1.04)	0.06
BNP, per 10 pg/dl increase	1.00 (1.00–1.01)	<0.001
Creatine, per 0.1mg/dl increase	3.56 (1.07–11.9)	0.04
ECG data		
Heart rate, per 1 beats/min increase	1.00 (0.99–1.02)	0.63
QRS duration, per 10 msec increase	1.00 (0.99–1.01)	0.39
Echocardiography data		
LVEF, per 1% unit decrease	1.00 (0.99–1.01)	0.84
LVEDV, per 10 ml increase	1.00 (0.99–1.01)	0.64
LVESV, per 10 ml increase	1.00 (0.96–1.04)	0.47
^123^I-MIBG scintigraphy data		
delayed H/M, per 0.01 decrease	2.56 (1.13–5.88)	0.02
WR ≥ 45%	1.03 (1.00–1.06)	0.03
Presence of LGE	2.96 (1.31–6.69)	<0.001

HR, hazard ratio; CI, confidence interval; WR, washout rate; other abbreviations are as defined in Table 1.

**Table 3:** Multivariate Cox regression analyses for predicting outcome.

	Multivariable (Model 1)		Multivariable (Model 2)		Multivariable (Model 3)		Multivariable (Model 4)		Multivariable (Model 5)		Multivariable (Model 6)		Multivariable (Model 7)	
	HR (95% CI)	p Value	HR (95% CI)	p Value	HR (95% CI)	p Value	HR (95% CI)	p Value	HR (95% CI)	p Value	HR (95% CI)	p Value	HR (95% CI)	p Value
NYHA functional class	1.19 (0.62–2.28)	0.6	1.22 (0.64–2.33)	0.55	1.26 (0.66–2.40)	0.48	1.24 (0.66–2.38)	0.5						
Systolic BP, per 10 mmHg decrease	0.98 (0.96–1.00)	0.05							0.99 (0.97–1.01)	0.11	0.99 (0.97–1.01)	0.1	0.99 (0.97–1.01)	0.1
BNP, per 10 pg/dl increase	1.00 (1.00–1.01)	0.04	1.00 (1.00–1.01)	0.03	1.00 (1.00–1.01)	0.04	1.00 (1.00–1.01)	0.04	1.00 (1.00–1.00)	0.01	1.00 (1.00–1.00)	<0.01	1.00 (1.00–1.00)	<0.01
Creatinine,per 0.1mg/dl increase	2.22 (0.74–6.67)	0.16	2.27 (0.76–6.80)	0.14	2.30 (0.77–6.85)	0.14	2.28 (0.76–6.81)	0.14	1.11 (0.56–2.23)	0.76	1.11 (0.56–1.85)	1	1.02 (0.54–1.89)	0.96
WR ≥ 45%	3.88 (1.51–9.95)	<0.01							3.14 (1.09–9.06)	0.03				
Presence of LGE	2.40 (1.04–5.49)	0.04							4.06 (1.57–10.24)	<0.01				
WR<45%+LGE positive			1.83 (0.56–6.04)	0.32							0.89 (0.23–3.46)	0.86		
WR≥45%+LGE negative					0.77 (0.22–2.65)	0.69							1.25 (0.29–5.30)	0.77
WR≥45%+LGE positive			4.71 (1.8–12.3)	<0.01	3.18 (1.36–7.45)	<0.01	3.53 (1.75–7.15)	<0.01			4.09 (1.41–11.91)	<0.01	4.71 (1.84–12.04)	<0.01

All abbreviations are as defined in Table 1.

## Discussion

The main results are as follows: (Ⅰ) The findings of ^123^I-MIBG scintigraphy, such as delayed H/M and WR are not associated with the LGE status. (Ⅱ) The combination of WR by ^123^I-MIBG scintigraphy and LGE on CMR in DCM patients is a relevant prognostic marker of long-term cardiac events compared with either the presence of LGE or WR by ^123^I-MIBG scintigraphy alone. This study is the first clinical study to demonstrate that the combination of WR by ^123^I-MIBG scintigraphy and LGE on CMR serves as a strong predictor of very long-term outcomes in DCM patients.

### The combination of LGE on CMR and WR by ^123^I-MIBG scintigraphy

Detection of myocardial fibrosis was useful for the risk stratification of DCM patients, because increasing amount of fibrosis was associated with increased LV stiffness and reduced LV relaxation, and resulted in systolic dysfunction [15]. In the recent meta-analysis, LGE on CMR in DCM patients was associated with the increased risk of all-cause mortality, HF hospitalization [16] and life-threatening arrhythmic events [17]. However another study reported that no differences were found between patients with or without focal fibrosis detected with LGE and the amount of collagen volume fraction detected by endomyocardial biopsy [18]. LGE on CMR was the most accurate method to measure myocardial replacement fibrosis, but it was limited for the assessment of the diffuse interstitial fibrosis, which was commonly found in DCM patients [2]. Therefore, to avoid missing the high risk patients for cardiac events who had the diffuse intestinal fibrosis which cannot be identified with LGE, we added ^123^I-MIBG scintigraphy. ^123^I-MIBG scintigraphy showed myocardial uptake, storage and release mechanisms similar to those of plasma norepinephrine in the cardiac sympathetic nerve terminal. It had properties that allow its use for the noninvasive monitoring of the characteristics of cardiac sympathetic nervous function and of the myocardial β-adrenergic receptor signaling pathway, those were not detected by evaluation of myocardial properties, such as LGE on CMR [19–24].

In the HF status, the rein-angiotensin-aldosterone system was activated and the angiotensinⅡinduced expression of plasma connective tissue growth factor (CTGF) which was a cytokine that played a key role in the prognosis fibrosis, and expression of transforming growth factor-β(TGF-β) which caused cardiac fibrosis [25,26]. Moreover previous studies reported that, in DCM patients, these serum levels were strongly related with cardiac sympathetic nervous activity expressed by WR on ^123^I-MIBG scintigraphy [27,28]. In this study, regardless of the presence or absence of LGE, the median of WR was higher than the control [14] both groups, however, WR was not significantly different between the presence and absence of LGE. Thus, we divided the patients into four groups according to the presence or absence of LGE and the WR cut-off value for predicting prognosis based on ROC curve analysis. Indeed, WR ≥45%+LGE negative groups had the tendency of higher events rate than the WR<45% + LGE positive groups (Fig 5). Moreover the incidence of cardiac events was the highest in the extensive overlap of LGE areas and regional sympathetic nervous dysfunction of MIBG group (data not shown).Therefore, the combination of LGE on CMR and WR by ^123^I-MIBG scintigraphy provides more detailed risk stratification in DCM patients than LGE on CMR alone.

Momose et al. reported a 10-year long-term prognostic evaluation in DCM patients by ^123^I-MIBG scintigraphy, where the first 3-year survival curve with WR≥50% showed frequent cardiac events, but very few events during the rest of the follow-up periods [29]. However in our study, cardiac events occurred gradually over the 10 years follow-up period in high WR (WR≥45%) patients, the survival curve pattern was not similar to their study. In our study, 146 (99%) patients had received β-blockers at the time of undergoing ^123^I-MIBG scintigraphy, however in their study, only 24 (28%) patients received them. Compared with their study, almost all the patients receives adequate optimal medical therapy at the time of undergoing ^123^I-MIBG scintigraphy in our study, so we obtained more comprehensive information for assessing the risk of cardiac events.

### Limitation

This study is limited by the fact that it was a single center, retrospective study and had a small number of events, which might diminish the power of the drawn statistical inference. An additional limitation is that inappropriate cases of LGE on CMR and of ^123^I-MIBG scintigraphy could not be included in this study; thus, our conclusions might not be extrapolated to all DCM patients. Furthermore we did not assess LGE quantification and T1 mapping, because there is no current consensus on these techniques in DCM patients [2].Finally, we decided to use WR rather than early H/M and delayed H/M ratio for selecting different subgroup of patients, because, according to the ROC curve analysis for developing cardiac events, the area under the ROC curve for the WR was 0.68, however the area under the ROC curve for the early H/M and delayed H/M were smaller than WR,0.55 and 0.60, respectively.

## Conclusion

In DCM patients, the combination of WR by ^123^I-MIBG scintigraphy and LGE on CMR, which have different properties, might be useful to stratify the future cardiac events than LGE on CMR alone.
